# Stable Gastric Pentadecapeptide BPC 157 Therapy for Monocrotaline-Induced Pulmonary Hypertension in Rats Leads to Prevention and Reversal

**DOI:** 10.3390/biomedicines9070822

**Published:** 2021-07-15

**Authors:** Mario Udovicic, Marko Sever, Lovro Kavur, Kristina Loncaric, Ivan Barisic, Diana Balenovic, Gordana Zivanovic Posilovic, Dean Strinic, Sandra Uzun, Lovorka Batelja Vuletic, Suncana Sikiric, Anita Skrtic, Domagoj Drmic, Alenka Boban Blagaic, Martina Lovric Bencic, Sven Seiwerth, Predrag Sikiric

**Affiliations:** 1 Department of Pharmacology, School of Medicine, University of Zagreb, Salata 11, P.O. Box 916, 10000 Zagreb, Croatia; mario.udovicic@gmail.com (M.U.); dr.sever.marko@gmail.com (M.S.); lokavur@gmail.com (L.K.); kristinaloncaric@gmail.com (K.L.); inbarisic@gmail.com (I.B.); diana.balenovic@gmail.com (D.B.); gordana1709@gmail.com (G.Z.P.); destrinic@gmail.com (D.S.); sandra.uzun@vip.hr (S.U.); iddrmic@mef.hr (D.D.); abblagaic@mef.hr (A.B.B.); miminamama@gmail.com (M.L.B.); 2 Department of Pathology, School of Medicine, University of Zagreb, Salata 11, P.O. Box 916, 10000 Zagreb, Croatia; lbatelja@mef.hr (L.B.V.); suncanasikiric@gmail.com (S.S.); sven.seiwerth@mef.hr (S.S.)

**Keywords:** monocrotaline, pentadecapeptide BPC 157, rat, pulmonary arterial hypertension

## Abstract

**Background.** Monocrotaline selectively injures the lung’s vascular endothelium and induces pulmonary arterial hypertension. The stable gastric pentadecapeptide BPC 157 acts as a prototype cytoprotective agent that maintains endothelium, and its application may be a novel therapy. Besides, BPC 157 prevents and reverses thrombosis formation, maintains platelet function, alleviates peripheral vascular occlusion disturbances, and has anti-arrhythmic and anti-inflammatory effects. Monocrotaline-induced pulmonary arterial hypertension in rats (wall thickness, total vessel area, heart frequency, QRS axis deviation, QT interval prolongation, increase in right ventricle systolic pressure and bodyweight loss) can be counteracted with early or delayed BPC 157 therapy. **Methods and Results.** After monocrotaline (80 mg/kg subcutaneously), BPC 157 (10 μg/kg or 10 ng/kg, days 1–14 or days 1–30 (early regimens), or days 14–30 (delayed regimen)) was given once daily intraperitoneally (last application 24 h before sacrifice) or continuously in drinking water until sacrifice (day 14 or 30). Without therapy, the outcome was the full monocrotaline syndrome, marked by right-side heart hypertrophy and massive thickening of the precapillary artery’s smooth muscle layer, clinical deterioration, and sometimes death due to pulmonary hypertension and right-heart failure during the 4th week after monocrotaline injection. With all BPC 157 regimens, monocrotaline-induced pulmonary arterial hypertension (including all disturbed parameters) was counteracted, and consistent beneficial effects were documented during the whole course of the disease. Pulmonary hypertension was not even developed (early regimens) as quickly as the advanced pulmonary hypertension was rapidly attenuated and then completely eliminated (delayed regimen). **Conclusions.** Thus, pentadecapeptide BPC 157 prevents and counteracts monocrotaline-induced pulmonary arterial hypertension and cor pulmonale in rats.

## 1. Introduction

Due to its effects on endothelium maintenance and blood vessels (reviewed [1,2,3]), we focused on the therapeutic abilities of the stable gastric pentadecapeptide BPC 157 (reviewed [1,2,3,4,5,6,7,8,9,10,11,12,13,14]). As a cytoprotective agent (reviewed [4,5,6]), it may be a suitable form of treatment for pulmonary arterial hypertension, a disease characterized by progressive pulmonary vascular remodeling, resulting in right-side heart failure and premature death in rats given monocrotaline (reviewed [15,16]). In particular, monocrotaline is known to selectively injure the vascular endothelium of the lung and induces pulmonary vasculitis [17,18].

Before our study, pulmonary arterial hypertension and monocrotaline-induced pulmonary hypertension in rats had not been investigated (see [15,16] for reviews) from the viewpoint of the cytoprotection theory and concept (reviewed [4,5,6]). The cytoprotection concept holds that in the rat stomach, the rapid effects of endothelium lesions and endothelium maintenance are keys for the further cytoprotective therapy, which may have pleiotropic beneficial effects (reviewed [4,5,6]). As a novel anti-stomach-ulcer peptide that is stable in human gastric juice, BPC 157 is thought to be a novel mediator of stomach-based cytoprotection (reviewed [4,5,6]) (tested in an ulcerative colitis trial and now multiple sclerosis; lethal dose (LD1) not achieved) (reviewed [7,8,9]). BPC 157 maintains prostaglandins function in the mucosal integrity (reviewed [10]) and maintains the endothelium’s integrity (reviewed [4,5,6]).

The rapid rescuing of close endothelium damage by cytoprotective agents is what the original stomach cytoprotection studies perceived as the cytoprotection concept being realized. There is a shared class of cytoprotective agents that they began with [19,20], but BPC 157 [20] stands out due to its pleiotropic beneficial effects (reviewed [1,2,3,4,5,6,7,8,9,10,11,12,13,14]). These compounds are meant to reverse the damage caused by monocrotaline. As pointed out in reviews [15,16], pulmonary vascular endothelial damage, within hours after injection, becomes endothelial damage and inflammatory infiltration. Edema results after 1 week. Two weeks after monocrotaline injection, pulmonary artery hypertension is present, which leads to right ventricle (RV) hypertrophy by the third week, and thereafter, death results for half of the injected rats [15,16]. We thought it likely that BPC 157 therapy would have two effects, prophylactic and curative. When it is used soon after monocrotaline injection, it may prevent the development of the monocrotaline-induced pulmonary hypertension with all its manifestations. Likewise, it may reverse already established monocrotaline-induced pulmonary hypertension when given as a postponed regimen. Those would be prophylactic and curative effects, respectively. We knew that if both effects can result from BPC 157 therapy, we may avoid the known problems of standard cytoprotection studies and standard cytoprotective agents (effective only “before”) [4,5,6]. Likewise, this therapy could prove superior to the standard agents used in pulmonary arterial hypertension therapy (more “prevention” than “treatment”) [21,22,23,24,25,26]. Additionally, considering its described effects (and the μg–ng range used) (reviewed [1,2,3,4,5,6,7,8,9,10,11,12,13,14]), we considered it possible that both beneficial effects would appear using two distinctive BPC 157 regimens: either given daily intraperitoneally or given per-orally in drinking water.

Important for the possible therapeutic effects in response to monocrotaline-induced pulmonary hypertension is that BPC 157 directly protects the endothelium [20,27]. There is evidence that BPC 157 prevents and reverses thrombosis formation after abdominal anastomosis and the occlusion of major vessels [27,28,29,30], and that it alleviates the peripheral vascular occlusion disturbances [27,28,29,30,31,32,33,34,35] while rapidly activating alternative bypassing pathways [28,29,30,31,32,33,34]. Evidence in favor of BPC 157 includes the recovery of the Pringle maneuver ischemia and reperfusion in rats (portal triad temporary occlusion), and the recovery of the Budd–Chiari syndrome rats (permanent occlusion of the suprahepatic inferior caval vein) [29,30]. The shared pathology of those diseases represents the right-heart problems that commonly appear [29,30]. Similarly, lung congestion appears as a common outcome (i.e., time-dependent and time-independent features that can be acute respiratory distress syndrome exudative phase features) [29,30]. Besides, BPC 157, as a part of its wound-healing effect (reviewed [11]), counteracts prolonged bleeding and thrombocytopenias (i.e., in the various models, i.e., anticoagulants, amputation of the tail or leg [36,37], and or organ perforation [33]). Additionally, BPC 157 maintains platelet function [38]. In regard to healing angiogenesis (reviewed [11]), the stable gastric pentadecapeptide BPC 157 is superior to the standard angiogenic factors, and its curative effect is a result of its interactions with several molecular pathways [3,13,14,28,35,39,40,41,42,43,44].

Finally, besides the prostaglandins system (reviewed [10,14]), there is evidence that BPC 157 beneficially affects also both ET-1 and NO systems (reviewed [3]). This should be favorable in consideration of the specific therapies developed for pulmonary arterial hypertension (reviewed [15,16]). It targets three signaling pathways, endothelin-1, prostacyclin, and nitric oxide (NO) pathways (reviewed [15,16]). Note, monocrotaline intoxication goes along with increased intima expression of endothelin-1 [45] and decreased expression of endothelin receptors and eNOS [46,47]. Illustratively, preventing and reversing the increased expression of endothelin-1 makes up part of BPC 157’s cardioprotective therapy. Accordingly, there is the counteraction of doxorubicin’s chronic heart failure (both prevention and reversal) [48]. Besides, BPC 157’s interaction with the NO system is impactful (reviewed [3]). It occurs in various models and species (reviewed [3]), and BPC 157 counteracts the adverse effect of nitric oxide synthase (NOS)-blocker much like that of NOS-substrate [3,32,33,34,49,50,51,52] (including the counteraction of a particular kind of pulmonary arterial hypertension (ascites) in chickens caused by an inability to synthesize L-arginine [51]). BPC 157 may induce the release of NO on its own [52,53], even when L-arginine is not present [53], and stimulates eNOS in the vessels during occlusion [29]. Accordingly, BPC 157 directly affects potassium conductance [54,55,56] and sodium channels [57], and counteracts various arrhythmias [30,31,54,56,57,58,59,60,61]. This counteraction involves, in particular, a prolonged QTc interval [30,59], a thing correlated with pulmonary pressures and right-ventricular dilation, and inversely correlated with RV function [62].

Thus, considering that in pulmonary arterial hypertension the RV and pulmonary circulation constitute a unified cardiopulmonary unit and that the RV can be therapeutically targeted in pulmonary arterial hypertension [62], this BPC 157 monocrotaline study in rats provides strong evidence for both prevention and counteraction of the monocrotaline-induced pulmonary hypertension and cor pulmonale.

## 2. Methods

### 2.1. Animals

The study was conducted with male Albino Wistar rats, bodyweight 200 g, 13 weeks old, in-house bred at the animal facility of the Department of Pharmacology, School of Medicine, Zagreb, Croatia. The animal facility was registered by Directorate of Veterinary Sciences, registration number HR-POK-007. Laboratory rats were acclimated for 5 days and randomly assigned to their respective treatment groups. Laboratory animals were housed in PC cages in conventional laboratory conditions with a temperature of 20–24 °C, relative humidity of 40–70%, and a noise level of 60 DCB. Each cage was identified by dates, the number of the study, group, dose, and the number and sex of each animal. Fluorescent lighting provided illumination 12 h per day. A standard GLP diet and fresh water was provided ad libitum. Animal care was in compliance with the SOPs of the Department of Pharmacology’s animal facility and the European conventions for the protection of vertebrate animals used for experimental and other scientific purposes (ETS 123). Ethical principles of the study ensured compliance with European Directive 010/63/E, the Law on Amendments to Animal Protection Act (Official Gazette 37/13, the Animal Protection Act (official Gazette 135/06), Ordinance on the protection of animals used for scientific purposes (Official Gazette 55/13), FELASA recommendations, and recommendations of the Ethics Committee, School of Medicine, University of Zagreb. All experiments received specific approval from the Local Ethics Committee at the School of Medicine (University of Zagreb, Zagreb, Croatia). We randomly assigned 10 rats per experimental group and per period for all experiments.

### 2.2. Drugs

Monocrotaline (Sigma, St. Louis, MI, USA) was diluted in PBS. We applied pentadecapeptide BPC 157 (manufactured by Diagen, Ljubljana, Slovenia, GEPPPGKPADDAGLV, M.W. 1419), a partial sequence of human gastric juice protein BPC and a peptide with 99% (HPLC) purity which is freely soluble in water at pH 7.0 and in saline, dissolved in saline; dose ranges and ways of application as previously described (reviewed [1,2,3,4,5,6,7,8,9,10,11,12,13,14]).

### 2.3. Protocol

**Figure 1:**
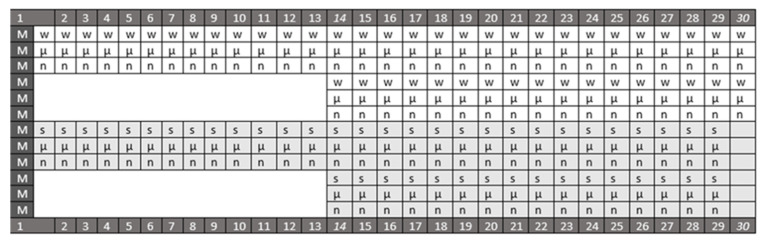
Shematic presentation of the given protocol. Dark gray rows (white letters) indicate days (1–30; 14 and 30 indicate days of sacrifice, or later initiation of the therapy) after monocrotaline (M) (80 mg/kg subcutaneously on day 1, as described before [17]). Stable gastric pentadecapeptide BPC 157 medication (10 μg/kg (μ) or 10 ng/kg (n)) (as reviewed in [1,2,3,4,5,6,7,8,9,10,11,12,13,14]) was given intraperitoneally (light gray rows) or per-orally in drinking water (white rows); controls received an equal volume of saline (5 mL/kg intraperitoneally) (s) or drinking water (w).

Monocrotaline was given as a single subcutaneous injection in an 80 mg/kg dose, as described before [17]. The stable gastric pentadecapeptide BPC 157 medication was 10 μg/kg or 10 ng/kg therapy (as reviewed [1,2,3,4,5,6,7,8,9,10,11,12,13,14]) (Figure 1). Early therapy, the BPC 157 prophylactic regimen, included the first intraperitoneal application immediately after monocrotaline, and the last 24 h before sacrifice; or application per-orally, 0.16 μg/mL/rat/day or 0.16 ng/mL/rat/day continuously in drinking water. The delayed application of the BPC 157 therapeutic regimen started after monocrotaline-induced pulmonary arterial hypertension had been already established. The first intraperitoneal application was on day 14, and the last was 24 h before sacrifice. The per-oral application (0.16 μg/mL/rat/day or 0.16 ng/mL/rat/day continuously in drinking water) began on day 14 and continued till sacrifice. Simultaneously, controls received an equal volume of saline 5 mL/kg intraperitoneally one time daily, or drinking water only (12 mL/rat/day), till sacrifice at the day 14 or day 30. The animals were sacrificed by stunning and cervical dislocation [21].

### 2.4. Echocardiography

Deeply anesthetized rats (ketamine 40 mg/kg, i.p. plus diazepam 10 mg/kg, i.p.) were laid on their backs under anesthesia with diethyl ether. Two-dimensional echocardiography was performed with an echocardiographic system (Philips SD800, MA, USA) and a 7.5-MHz linear probe on day 1 prior to monocrotaline application; days 7, 14, and 21; and on day 30, before sacrifice. In the parasternal echocardiographic window, a two-dimensional short-axis view of the left ventricle was obtained at the level of the papillary muscle. To estimate the increase in right ventricle systolic pressure, we calculated the ratio of the minor axis to the major axis of the left ventricle in the end-systolic phase, as previously described [63]. Measurements were performed by a single observer naive about the treatment. The development of pulmonary hypertension was monitored non-invasively by echocardiography, since it is less aggressive and painful while showing a high degree of correlation with magnetic resonance imaging or direct cardiac catheterization [63,64]. ECG was recorded continuously for anesthetized rats by positioning stainless steel electrodes on all four limbs, using an ECG monitor by 2090 Medtronic programmer (Minneapolis, MN, USA).

### 2.5. Microscopy

On day 30 the rats were sacrificed. The lungs were removed and embedded in paraffin after fixing by 4% paraformaldehyde.

Parts of the lungs and hearts were harvested, fixed with 4% paraformaldehyde, and embedded in paraffin for morphological analysis. Sections (3–4 μm) were stained with the orcein–van Gieson method. Pulmonary arterioles with an external diameter (ED) approximately 50–150 μm were chosen for morphological analysis. Total vascular area (TA) and lumen area (LA) of the pulmonary arterioles were determined by image analysis software ISSA (VAMSTECH, Zagreb) quantitatively. The percentage of wall area (WA) to the total area of vessels (WA%, WA% = (TA − LA)/TA × 100%) was calculated.

The right ventricle (RV) and left ventricle plus septum (LV + S) were weighed as previously described [65], and the right-ventricular hypertrophy index was calculated as the weight ratio of RV/(LV + S) and used to describe the degree of right-ventricular hypertrophy.

### 2.6. Statistical Analysis

Statistical analysis was performed using Statistica 7.0. parametric. A Kolmogorov–Smirnov test was used to assess normal distribution of data. If normally distributed, one-way ANOVA with a post-hoc Tukey–Kramer test was used. A Kruskal–Wallis test with a post-hoc Mann–Whitney U test was used for non-normal distribution. Proportions were analyzed by Fisher’s exact test. The differences were considered statistically significant if *p* < 0.05.

## 3. Results

Without active therapy, monocrotaline-rats presented a downhill course. There was both severe monocrotaline syndrome and 50% mortality in the controls at the end of the experiment (*p* ˂ 0.05 vs. BPC 157 (without mortality), Fisher’s exact probability test). With BPC 157, disease was completely lacking (prophylactic regimen). If present, it was quickly attenuated and finally eliminated (delayed application regimen). A comparable beneficial effect was obtained with intraperitoneal application and application in the drinking water, with μg and ng regimens.

As a sign of heart failure comparable to cachexia in patients with chronic heart failure, the bodyweights of all controls over a 4-week period after monocrotaline application indicated pronounced growth retardation and loss of weight during week 4. Contrarily, all BPC treated animals maintained normal bodyweights. Additionally, all control rats showed a lack of physical activity in contrast to the BPC 157-treated rats’ normal behavior (Table 1 and Table 2). Furthermore, unlike BPC 157-treated rats, all control animals had increased liver weights, indicating liver congestion as a consequence of right-heart failure.

**Figure 2:**
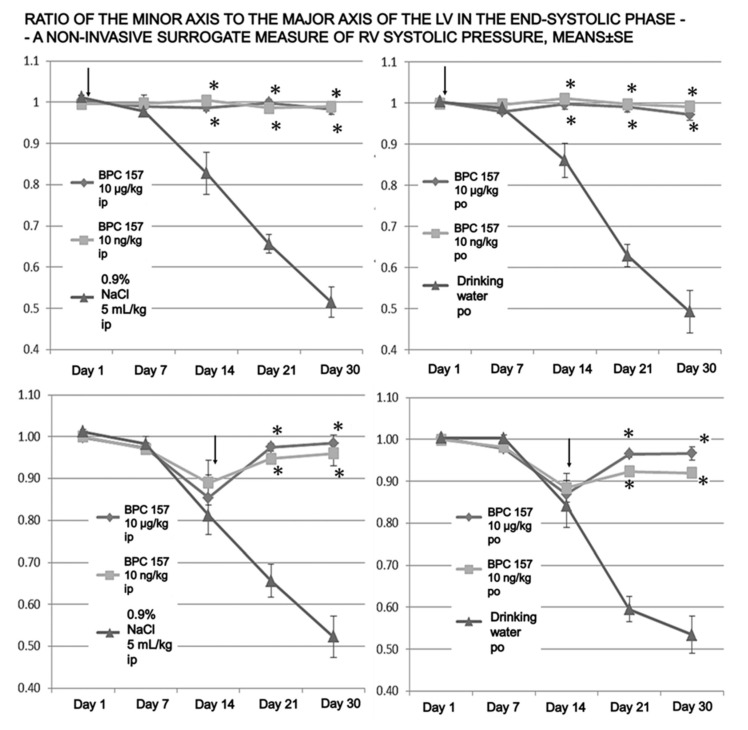
Time course of pulmonary hypertension evaluated by two-dimensional echocardiography. Changes in the ratio of the minor axis to the major axis of the LV in the end-systolic phase—as a non-invasive surrogate measure of RV systolic pressure. The decrease in the ratio in-group was inhibited or prevented by BPC 157 application in all treatment groups, showing significance already on day 21. Arrows indicate initiation of the therapy (prophylactic or curative). * *p* ˂ 0.05 at least vs. control.

Indicatively, the ratio of the minor axis to the major axis of the LV in the end-systolic phase in pulmonary arterial hypertension in control rats was downhill from day 7 on, and severely deteriorated at the latter periods, i.e., at days 14, 21, and 30 (Figure 1 and Figure 2). With the prophylactic regimen—the therapy was given continuously from the very beginning—this ratio’s deterioration was completely avoided, and the ratio remained constant; it did not deteriorate after the monocrotaline challenge.

For the therapy given continuously from day 14 onward, starting after the established ratio deterioration, the deterioration halted within one week, as assessed on day 21, and the ratio then remained constant, and did not deteriorate until the end of the experiment (i.e., day 30) (Figure 1 and Figure 2).

**Figure 3:**
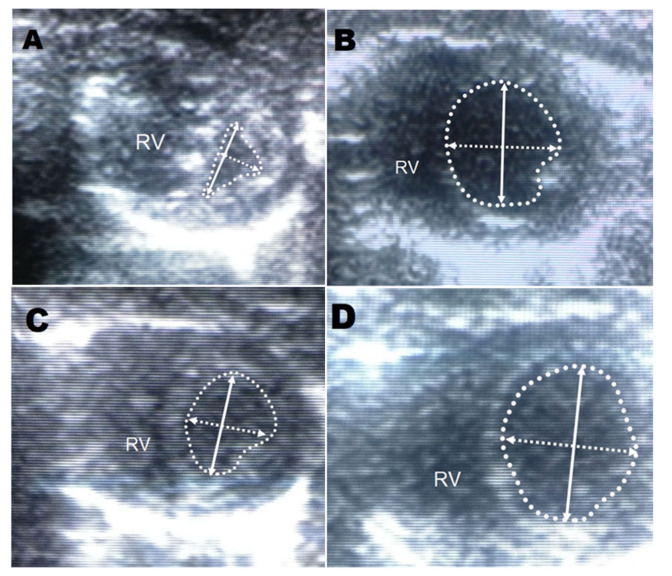
Short axis echocardiography: (**A**). A monocrotaline (control) rat (0.9% NaCl 5 mL/kg i.p.) on day 30 with a dilated right ventricle due to the high pulmonary pressure and pronounced deformation of the left ventricle. There is a reduced ratio of the minor axis of the left ventricle to the major axis (**upper, left**). (**B**). The prophylactic BPC 157 regimen (BPC 157 10 μg/kg i.p.) on day 30 with a normal left ventricle (**upper, right**). (**C**). Monocrotaline rats with no intervention on day 14 (**upper, left**). (**D**). Delayed BPC 157 application (BPC 157 10 μg/kg i.p.) on day 30 (**upper, right**). (RV—right ventricle (RV), solid arrow—major axis of the left ventricle (LV), dashed arrow—minor axis of LV, dotted line—LV contour).

**Figure 4:**
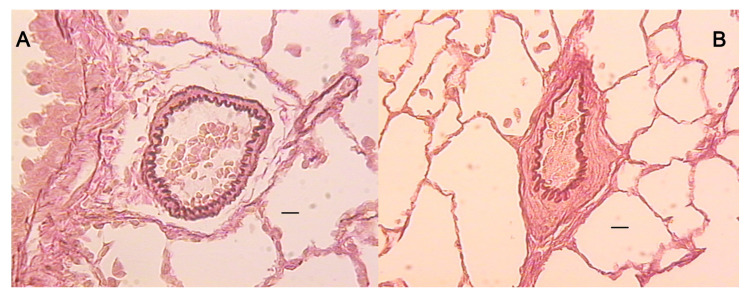
Orcein–van Gieson staining of the pulmonary artery in the BPC 157 therapy group, 10 μg/kg po (**A**, ×10), and the corresponding control group (**B**, ×10). The lines indicate 50 µm. The walls in the control rats were thicker than those in the treatment groups.

**Figure 5:**
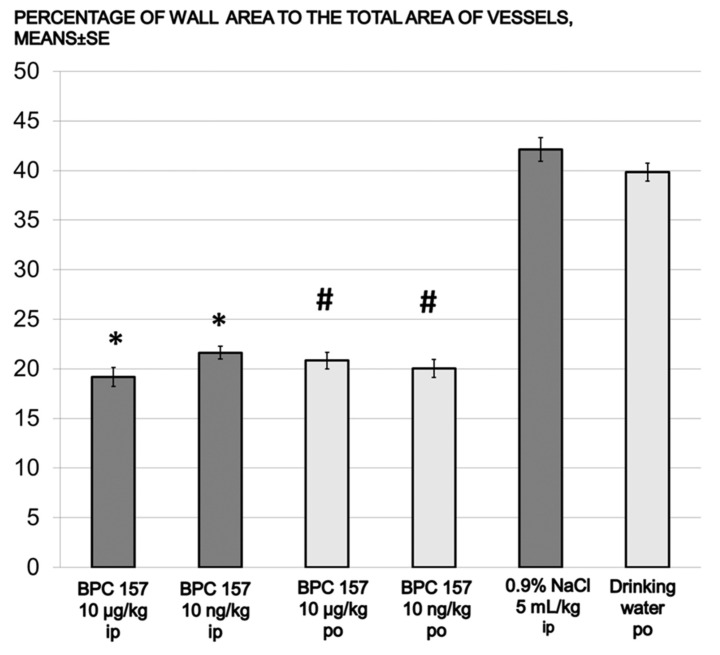
Prophylactic regimen. Percentage of wall area to the total area of vessels for prophyactic groups (means ± SE), BPC 157 10 μg/kg or 10 ng/kg. Medication: one time daily intraperitoneally; first application immediately after monocrotaline, last 24 h before sacrifice (dark gray bars). Medication: BPC 157, 0.16 μg/mL/rat/day or 0.16 ng/mL/rat/day continuously in drinking water until sacrifice (day 30) (light gray bars), * *p* < 0.05 vs. 0.9%NaCl i.p. (dark gray bars), # *p* < 0.001 vs. drinking water po (light gray bars).

**Figure 6:**
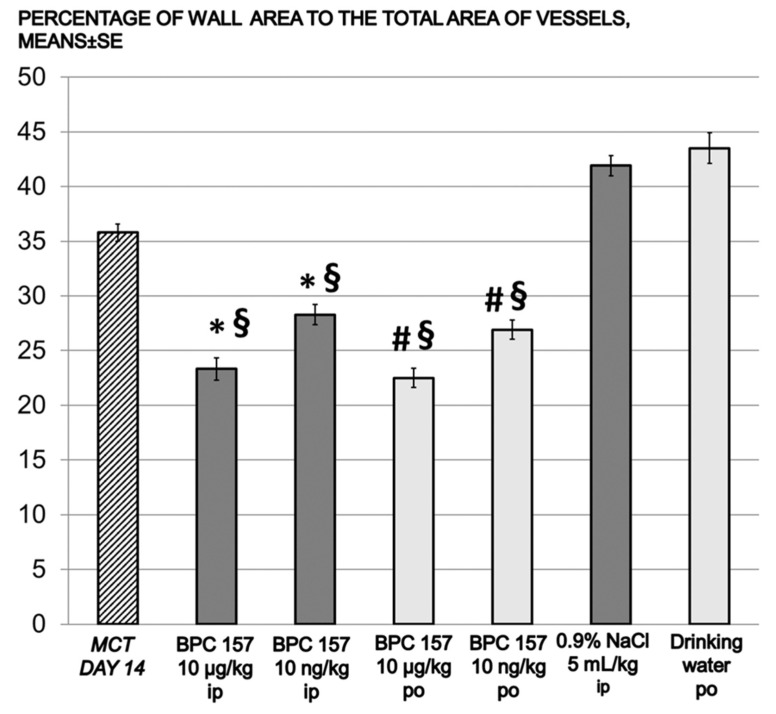
Delayed application. Percentage of wall area to the total area of vessels in monocrotaline rats (means ± SE); BPC 157 10 μg/kg or 10 ng/kg. Medication: one time daily, intraperitoneally; first application on day 14 after monocrotaline, last 24 h before sacrifice (dark gray bars). Medication: BPC 157 0.16 μg/mL/rat/day or 0.16 ng/mL/rat/day continuously in drinking water) from day 14 until sacrifice (light gray bars). § *p* < 0.05, at least, vs. monocrotaline (MCT) on day 14 (dashed bar); * *p* < 0.05 vs. 0.9% NaCl i.p. (dark gray bars), # *p* < 0.05, at least, vs. drinking water po (light gray bars).

All controls showed significantly lower heartbeat frequencies, prolonged QT intervals, and marked deviations in the QRS axis to the right by day 14, all of which progressed until day 30. All BPC 157 groups presented undisturbed heart frequencies and QT intervals, and no deviation in the QRS axis to the right, as of day 30. Again, this was the case for the prophylactic regimen and the delayed regimen (rescue of the previous disturbances). Although LV and interventricular septum (IVS) weights were unaffected from day 14 on, the RV weight in controls markedly increased. BPC 157 therapy (both regimens) resulted in undisturbed RV weight (and thereby, on day 14, immediate counteraction of the ongoing disturbances came about). The degree of RV hypertrophy was determined as the RV/(LV + IVS) weight ratio, and the RVs remained unchanged in all BPC 157-treated rats (Table 1 and Table 2). In controls, the monocrotaline-induced pulmonary arterial hypertension occurred, along with the increased wall area and marked hypertrophy of arterial media already at day 14, sustained till the end of the experiment (day 30) in all controls. Likewise, along with the mentioned therapeutic effect, all BPC 157 rats had undisturbed wall areas and no hypertrophy of arterial media on day 30 (and thereby, the rescue of the previous disturbances, positive reverse remodeling, and reduction due to the given BPC 157 medication) (Figure 3, Figure 4, Figure 5 and Figure 6).

## 4. Discussion

This study attempted to resolve monocrotaline-induced pulmonary arterial hypertension in rats (i.e., initial pulmonary vascular endothelial damage, pulmonary arterial hypertension, RV hypertrophy, and fatality) (reviewed [15,16]), from the viewpoint of stomach cytoprotection (i.e., in the damaged rat stomach, rapid endothelium maintenance is the key for a pleiotropic beneficial effect) (reviewed [4,5,6]). Thereby, against monocrotaline-induced pulmonary arterial hypertension, BPC 157 therapy stands as a cytoprotective agent prototype (reviewed [4,5,6]). The complete severe monocrotaline syndrome course was markedly affected. With therapy, disease development was completely lacking (prophylactic regimen). Likewise, when monocrotaline syndrome was in an advanced stage, it was quickly attenuated and finally eliminated (delayed application regimen). Such beneficial effects were also obtained with the daily intraperitoneal injections and administration in drinking water (since it is stable in human gastric juice for more than 24 h (reviewed [1,2,3,4,5,6,7,8,9,10,11,12,13,14])), and with μg and ng regimens (reviewed [1,2,3,4,5,6,7,8,9,10,11,12,13,14]). Together, this means a consistent effect and a wide range of options for administration (i.e., bolus (intraperitoneal) vs. small amounts taken ad libitum (therapy given in the drinking water)). It appears to very nearly cause the practical elimination of monocrotaline’s toxicity and pulmonary arterial hypertension as a common outcome.

Of note, the indicated general background of the BPC 157 effects (cytoprotection, endothelium rebuilding, cardioprotection, antiarrhythmic potential, providing a safe profile, and preventing LD1 from being achieved) (reviewed [4,5,6]) may include additional, more indicative support. RV hypertrophy and increased RV weight [22,23], along with QTc prolongation, appear in both human and animal studies of pulmonary hypertension [66,67,68,69] and in patients with pulmonary arterial hypertension as an independent predictor of mortality [66].

Thereby, BPC 157 regimens, showed cause–consequence significance in counteracting monocrotaline-induced pulmonary arterial hypertension and QTc interval elongation. Thus, along with previous studies [30,59], we presented experimental evidence that the RV can be therapeutically targeted in pulmonary arterial hypertension. Of note, the obtained evidence is complete (i.e., monocrotaline-treated rats develop significant pulmonary hypertension and marked RVe hypertrophy; development was effectively prevented with the early BPC 157 (prophylactic) regimens, and once established, was fully counteracted by BPC 157 administration (therapeutic regimen)). Likely, this may resolve the traditional view (the RV dysfunction of monocrotaline-treated rats as a direct consequence of pressure overload) [62]. BPC 157 counteracts the right-heart disturbance and the lung congestion course that commonly appears in the Pringle maneuver ischemia and reperfusion rats and in the Budd-Chiari syndrome rats [29,30]. This was in addition to the counteracted elongation of the QT interval and the known antiarrhythmic effect [30,31,54,56,57,58,59,60,61].

We have ascertained a beneficial effect, but the full mechanism remains elusive. It may be that BPC 157 more effectively interferes with the essential mechanisms of monocrotaline’s toxicity [70]. As mentioned before [15,16], the first effect is pulmonary endothelial cells being initially injured [71,72]. The next effects may be smooth muscle cell proliferation, endothelial cell mesenchymal transition, and endothelial cell dysfunction as a consequence of non-pulmonary arterial endothelial cell damage [71,72]. These effects are likely counteracted by BPC 157, considering its cytoprotective properties (reviewed [4,5,6]). BPC 157 directly protects endothelium [20,27], prevents and reverses thrombosis formation [27,28,29,30], and maintains platelets function [38]. Finally, BPC 157 alleviates peripheral vascular occlusion disturbances [27,28,29,30,31,32,33,34,35], rapidly activating alternative bypassing pathways [28,29,30,31,32,33,34]. Alternatively, the first pathogenic effect may be systemic inflammation triggered by monocrotaline exposure [21]. Illustrating its anti-inflammatory potential, BPC has been shown to counteract tumor-cachexia-induced muscle wasting [13]. It counteracts the increases of pro-inflammatory and pro-cachectic cytokines, such as IL-6 and TNF-α, and the expression of FoxO3a, p-AKT, and P-GSK-3β. Note, skeletal muscle wasting is also postulated to be related to pulmonary arterial hypertension [73]. BPC 157 also acts as a membrane stabilizer [14]. It mitigated indomethacin-induced leaky gut syndrome via increasing tight junction protein ZO-1 expression and transepithelial resistance [14]. It inhibited the mRNA of inflammatory mediators (iNOS, IL-6, IFNγ, and TNF-α). On the other hand, it increased the expression of HSP 70 and 90, and antioxidant proteins such as HO-1, NQO-1, glutathione reductase, glutathione peroxidase 2, and GST-pi [14]. The third possibility is monocrotaline accumulation in the erythrocytes [74,75,76]. In the erythrocytes, monocrotaline conserves its capability to interact with lung tissue [74,75,76]. In support of this theory, significant changes in pulmonary arterial pressures, the medial thickness of small pulmonary arteries, and right ventricle hypertrophy do not occur until the 3rd or 4th week after monocrotaline exposure [77]. BPC 157 may counteract that additional delayed effect of the accumulated monocrotaline. The consistent beneficial effect verifies that BPC 157 beneficially acts at that time as well. Additionally, recently, by in situ hybridization and immunostaining, BPC 157 was found in human gastrointestinal mucosa and lung bronchial epithelium; therefore, it may have additional regulatory roles in the human gastrointestinal tract and lungs, which, however, remain to be determined [11]. 

On the other hand, these findings (i.e., the same beneficial effect of both the prophylactic regimen and the delayed therapeutic regimen) seem to be valuable also with respect to the model, and its limitations [21]. It was postulated that the clear evidence of pulmonary hypertension in the untreated monocrotaline group on day 14 meant the experimental protocol was comparable to the clinical situation [21]. Consequently, with respect to the worsened conditions, which have to be present at that time [21], the delayed therapy initiation (i.e., day 14) was well chosen [23] (note, shorter intervals (day 11 or day 12) after monocrotaline [21,24] may have been premature, and thereby misleading considering the therapeutic effect [21]). Additionally, all of the assessed disturbance parameters (which were all counteracted) were used in other studies [21]. They included disturbed wall thickness, total vessel area, and heart frequency; QRS axis deviation; QT interval prolongation; RV hypertrophy; increased right ventricle weight [22,78]; an increase in RV systolic pressure; mortality; and bodyweights loss. In particular, the reduced bodyweight as a marker of clinical deterioration in the animal, as in the patient, again accords with previous studies [22]. Likewise, with respect to the timing of the initiation of therapy being crucial [21], the prophylactic (just after monocrotaline) and therapeutic (on day 14 after monocrotaline) results fully matching is an excellent sign. 

Finally, the exact mechanism should be further determined considering the standard drugs (i.e., the endothelin receptor antagonists, phosphodiesterase-5 inhibitors, statins, beta blockers, and Rho kinase inhibitor) [21,22,23,24,25,26]. A greater effect is normally thought to result from “prevention” than “treatment” [21]. Combining anti-proliferative therapy (such as rapamycin) and proapoptotic agents (statins) [24] shares that same limitation (i.e., effective only early, but not after delaying treatment). Likewise, considering the studies (i.e., [23,79,80]) it seems [21] that early therapy with endothelin receptor antagonists may only delay rather than stop the development of pulmonary hypertension (whereas BPC 157 eliminated monocrotaline-induced pulmonary arterial hypertension). Note, a reduction in bodyweight, RV hypertrophy, and an increase in RV weight still occur when treatment involves a endothelin receptor antagonist (e.g., macitentan) [22,23,78], and thereby, the underlying pathogenesis of the monocrotaline model continues to progress, and pulmonary hypertension continues to develop despite the treatment [21], unlike the noted elimination in the BPC 157 regimens.

Thus, it is likely that BPC 157’s effects may be superior to those of the standard agents [21]. Illustratively, improved endothelial function and vasodilating properties, a reduction of smooth muscle hypertrophy within the media of the pulmonary vessels [79,80], and limiting the proinflammatory and proliferative effects of endothelin [21] are effects of the endothelin receptor antagonists. However, one should note the absence of effects when the circulating levels of endothelin have risen [21,80]. On the other hand, confronted with raised circulating levels of endothelin [48], BPC 157 cardioprotective therapy has a consistent beneficial effect [48]. The preventing of the endothelin increase goes along with the preventing of the doxorubicin-induced chronic heart failure [48]. Thus, reversal of the increase in circulating endothelin [48] occurs alongside the counteraction of the established doxorubicin chronic heart failure in mice and rats [48]. Likewise, concerning the endothelin/NO system relation [48], BPC 157’s interaction with the NO system occurs in various models and species (reviewed [3]). As a result, BPC 157 counteracts the adverse effect of an NOS-blocker much like an NOS-substrate [3,32,33,34,49,50,51,52]. Furthermore, BPC 157′s effect on endothelium, in addition to its strong interactions with the NO system (for a review, see [3]) is strongly supported by the most recent demonstration that BPC 157’s effect on vasomotor tone is carried out through BPC 157-specific activation of the Src–Caveolin-1–endothelial nitric oxide synthase (eNOS) pathway [81].

Thereby, we can suggest that this BPC 157-monocrotaline study in rats provides evidence of a consistent therapeutic effect that resolves monocrotaline-induced pulmonary hypertension and cor pulmonale. In consideration of the monocrotaline model’s significance and limitations (reviewed [15,16]), BPC 157 will likely be applicable for therapy.

## 5. Conclusions

To conclude, the present study favored the realization of the cytoprotection concept by using the stable pentadecapeptide BPC 157, as a prototypical cytoprotective agent (reviewed [4,5,6]), to cure pulmonary arterial hypertension. The mechanism has two initial targets (i.e., the lung endothelium (monocrotaline in pulmonary arterial hypertension)—reviewed [15,16]; the stomach endothelium (cytotoxic agents in the cytoprotection stomach studies [19,20])). BPC 157 functions post-monocrotaline-injury, providing BPC 157 easy applicability (intraperitoneally or per-orally, in drinking water, μg and ng regimen as in other studies (reviewed [1,2,3,4,5,6,7,8,9,10,11,12,13,14])), and it has a safe profile (LD1 was not achieved: reviewed in [1,2,3,4,5,6,7,8,9,10,11,12,13,14]). The therapeutic effect covers the complete course of monocrotaline-induced pulmonary hypertension: the development (early, prophylactic regimens; prophylactic effect) and the advanced, late stage (delayed regimens; curative effect). This suggests rapid cytoprotective rescue, which is complex in pulmonary arterial hypertension, as the earliest defense is also essential for the final outcome (reviewed [4,5,6]). Likely, it may be analogous to the innate rapid rescuing of the damaged endothelium with cytoprotective agents in the stomach lesion studies (reviewed [4,5,6]): quickly propagating towards reestablishing mucosal integrity (reviewed [4,5,6]). In such cases, regaining tissue integrity occurs quickly after the agent’s application (reviewed [4,5,6]). Thereby, the endothelium rescue provided by BPC 157 strongly opposes the chain of events that leads to RV failure and normalizes pulmonary arterial hypertension, even if it is already advanced, as we consistently found in experiments.

## Figures and Tables

**Table 1:** Therapy (BPC 157 10 µg/kg, 10 ng/kg) started immediately after monocrotaline (80 mg/kg sc) injection. Assessment on day 30; means ± SE. Prophylactic regimens. A. Medication: BPC 157 one time daily, intraperitoneally; first application immediately after monocrotaline (day 0), last 24 h before sacrifice. B. Medication: BPC 157 (0.16 µg/mL/rat/day or 0.16 ng/mL/rat/day) continuously in drinking water until sacrifice.

Assessed Parameters	Therapy Started Immediately after Monocrotaline Application					
	A. Medication: One Time Daily, Intraperitoneally * *p* ˂ 0.05, at Least, vs. Control			B. Medication: BPC 157 Continuously in Drinking Water until Sacrifice # *p* ˂ 0.05, at Least, vs. Control		
	0.9% NaCl 5 mL/kg	BPC 157 10 µg/kg	BPC 157 10 ng/kg i.p.	Drinking Water 12 mL/rat /day	BPC 157 10 µg/kg	BPC 157 10 ng/kg
BW end (g)	224.5 ± 3.46	252.5 ± 8.73 *	258.33 ± 4.59 *	226.75 ± 4.39	257.5 ± 5.28 #	243.67 ± 4.18 #
heart (g)	1.12 ± 0.04	0.88 ± 0.04	0.99 ± 0.06	1.19 ± 0.05	1.02 ± 0.09	0.95 ± 0.05
RV (g)	0.32 ± 0.03	0.15 ± 0.01 *	0.18 ± 0.01 *	0.34 ± 0.02	0.17 ± 0.01 #	0.17 ± 0.01 #
LV + IVS (g)	0.59 ± 0.03	0.53 ± 0.03	0.59 ± 0.05	0.62 ± 0.02	0.62 ± 0.06	0.58 ± 0.04
liver (g)	12.82 ± 0.43	8.72 ± 0.35 *	8.68 ± 0.36 *	12.3 ± 0.04	9.14 ± 0.44 #	9.90 ± 0.74 #
RV/(LV + IVS)	0.56 ± 0.07	0.29 ± 0.01 *	0.3 ± 0.01 *	0.54 ± 0.02	0.28 ± 0.01 #	0.29 ± 0.02 #
RV/BW (mg/g)	1.44 ± 0.14	0.61 ± 0.02 *	0.68 ± 0.03 *	1.42 ± 0.08	0.67 ± 0.04 #	0.68 ± 0.04 #
liver/BW(mg/g)	57.11 ± 3.76	34.6 ± 1.20 *	33.68 ± 1.63 *	52.37 ± 2.69	35.64 ± 2.07 #	40.66 ± 3.05 #
media area %	42.13 ± 1.19	19.19 ± 0.94 *	21.63 ± 0.66 *	39.83 ± 0.92	20.83 ± 0.84 #	20.04 ± 0.92 #
heart rate	319.2 ± 4.38	396.01 ± 1.46 *	392.8 ± 2.51 *	312.8 ± 2.68	400.4 ± 2.55 #	387.9 ± 3.36 #
respiration rate	139 ± 8.59	103.67 ± 1.74 *	102.67 ± 1.32 *	131 ± 7.41	102.67 ± 3.04 #	102.00 ± 2.01 #
QT interval	78 ± 2.32	44.67 ± 1.86 *	46.67 ± 1.68 *	83.58 ± 3.6	45.63 ± 2.09 #	48.96 ± 3.67 #

**Table 2:** Therapy (BPC 157 10 µg/kg, 10 ng/kg) started after monocrotaline (80 mg/kg sc) application on day 14. Assessment on day 14 (before therapy initiation) and on day 30 (end of the therapy); means ± SE. Therapeutic regimens. A. Medication: BPC 157 one time daily, intraperitoneally; first application day 14 after monocrotaline, last 24 h before sacrifice. B. Medication: BPC 157 (0.16 µg/mL/rat/day or 0.16 ng/mL/rat/day) continuously in drinking water from day 14 until sacrifice.

Monocrotaline (80 mg/kg sc, Day 0) Rats							
Assessed Parameters	Day 14 (before Therapy Initiation) § *p* < 0.05, at Least, vs. day 14	Day 30 (at the End of Therapy Protocol)					
		After Monocrotaline Application Therapy Started at Day 14					
		A. Medication: One Time Daily, Intraperitoneally * *p* ˂ 0.05, at Least, vs. Control			B. Medication: BPC 157 Continuously in Drinking Water Until Sacrifice # *p* ˂ 0.05, at Least, vs. Control		
		0.9% NaCl 5 mL/kg	BPC 157 10 µg/kg	BPC 157 10 ng/kg	Drinking Water 12 mL/rat /day	BPC 157 10 µg/kg water	BPC 157 10 ng/kg
BW end (g)	201.33 ± 6.4	207.6 ± 5.19	233.17 ± 9.24 *§	232.17 ± 5.15 *§	205.75 ± 3.20	240.17 ± 7.41 #§	232.33 ± 4.45 #§
heart (g)	0.77 ± 0.05	1.02 ± 0.04 §	0.85 ± 0.04	0.87 ± 0.03	1.04 ± 0.02 §	0.90 ± 0.05	0.93 ± 0.03
RV (g)	0.15 ± 0.01	0.26 ± 0.02 §	0.15 ± 0.01 *§	0.18 ± 0.01 *§	0.29 ± 0.01 §	0.16 ± 0.01 #§	0.19 ± 0.01 #§
LV + IVS (g)	0.41 ± 0.03	0.49 ± 0.01	0.52 ± 0.04	0.5 ± 0.02	0.5 ± 0.02	0.54 ± 0.03	0.55 ± 0.02
liver (g)	6.39 ± 0.44	12.85 ± 0.31 §	7.47 ± 0.58 *§	9.06 ± 0.33 *§	13.19 ± 0.22 §	8.19 ± 0.50 #§	8.47 ± 0.62 #§
RV/ (LV + IVS)	0.37 ± 0.02	0.53 ± 0.03 §	0.29 ± 0.01 *§	0.36 ± 0.02 *§	0.55 ± 0.02 §	0.29 ± 0.01 #§	0.34 ± 0.01 #§
RV/BW (mg/g)	0.75 ± 0.04	1.27 ± 0.07 §	0.64 ± 0.01 *§	0.77 ± 0.01 *§	1.39 ± 0.07 §	0.66 ± 0.01 #§	0.80 ± 0.01 #§
liver/BW (mg/g)	31.74 ± 1.83	62.07 ± 2.35 §	31.86 ± 1.54 *§	39.07 ± 1.44 *§	64.13 ± 0.99 §	34.05 ± 1.59 #§	36.43 ± 2.47 #§
media area %	35.79 ± 0.79	41.92 ± 0.93	23.33 ± 1.02 *§	30.15 ± 0.86	43.51 ± 1.40	22.58 ± 0.86 #§	28.50 ± 0.91 #§
heart rate	366.00 ± 2.73	316.02 ± 6.81 §	387.83 ± 4.59 *§	378.83 ± 6.74 *§	319.20 ± 7.14 §	375.50 ± 4.19 #§	379.17 ± 4.73 #§
respiration rate	114.67 ± 3.00	139 ± 8.59 §	98.67 ± 1.98 *§	100.7 ± 1.61 *§	131 ± 7.42 §	112.67 ± 2.81 #§	111.33 ± 1.91 #§
QT interval	61 ± 5.94	80 ± 3.82 §	48 ± 3.10 *§	52.7 ± 3.01 *§	89 ± 3.65 §	45.5 ± 2.75 #§	56.33 ± 0.76 #§

## Data Availability

The data presented in this study are available on request from the corresponding author.
